# Escape Velocity: Why the Prospect of Extreme Human Life Extension Matters Now

**DOI:** 10.1371/journal.pbio.0020187

**Published:** 2004-06-15

**Authors:** Aubrey D. N. J de Grey

## Abstract

Should we be considering the social and economic ramifications of a society where life-span could be limitless?

The biogerontologist David Sinclair and the bioethicist Leon Kass recently locked horns in a radio debate (http://www.theconnection.org/shows/2004/01/20040106_b_main.asp) on human life extension that was remarkable for one thing: on the key issue, Kass was right and Sinclair wrong. Sinclair suggested, as have other experts, including his mentor Lenny Guarente and the National Institute on Aging advisory council member Elizabeth Blackburn, that Kass and other bioconservatives are creating a false alarm about life extension, because only a modest (say, 30%) increase in human life span is achievable by biomedical intervention, whereas Kass's apprehensions concern extreme or indefinite life extension. Kass retorted that science isn't like that: modest success tends to place the bit between our teeth and can often result in advances far exceeding our expectations.[Fig pbio-0020187-g001]

**Figure pbio-0020187-g001:**
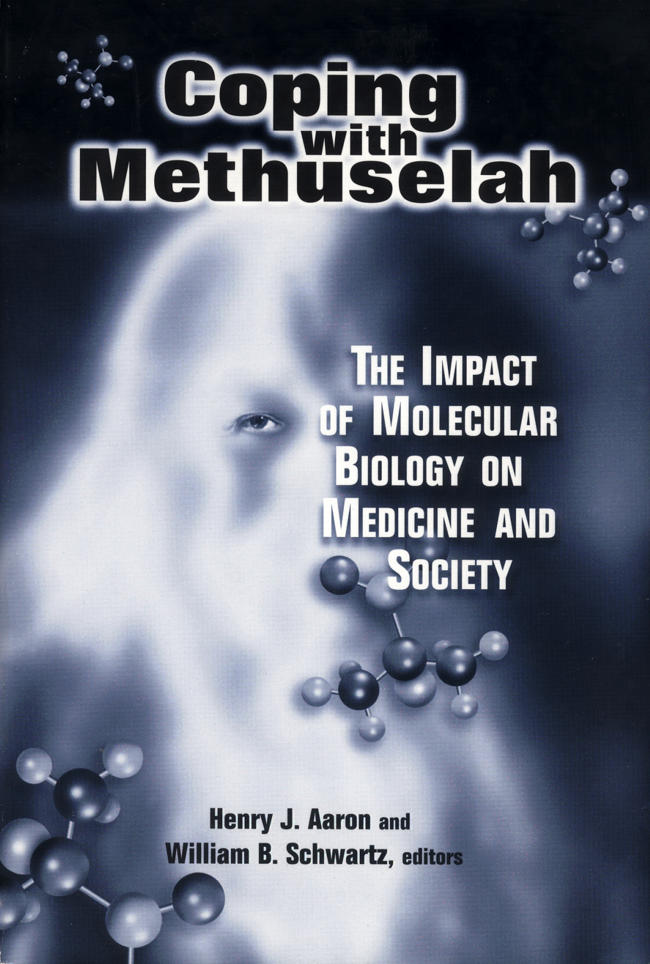


*Coping with Methuselah* consists of seven essays, mostly on the economics of life extension but also including one essay surveying the biology of aging and one on the ethics of life extension. The economic issues addressed are wide ranging, including detailed analysis of the balance between wealth creation by the employed and wealth consumption in pensions and health care; most chapters focus on the United States, but the closing chapter discusses these issues in a global context. Each essay is followed by a short commentary by another distinguished author. Within their own scope, all of these contributions are highly informative and rigorous. Dishearteningly, however, all echo Sinclair's views about the limited prospects for life extension in the coming decades. In my opinion, they make three distinct oversights.

The first concerns current science. Sinclair and several other prominent gerontologists are presently seeking human therapies based on the long-standing observation that lifelong restriction of caloric intake considerably extends both the healthy and total life span of nearly all species in which it has been tried, including rodents and dogs. Drugs that elicit the gene expression changes that result from caloric restriction might, these workers assert, extend human life span by something approaching the same proportion as seen in rodents—20% is often predicted—without impacting quality of life, and even when administered starting in middle age. They assiduously stress, however, that anything beyond this degree of life extension is inconceivable.

I agree with these predictions in two respects: that the degree of life extension achieved by first-generation drugs of this sort may well approach the (currently unknown) amount elicitable by caloric restriction itself in humans, and that it is unlikely to be much exceeded by later drugs that work the same way. In two other ways, however, I claim they are incorrect. The first error is the assumption of proportionality: I have recently argued (de Grey 2004), from evolutionary considerations, that longer-lived species will show a smaller maximal proportional life-span extension in response to starvation, probably not much more than the same *absolute* increase seen in shorter-lived species. The second error is the assertion that no other type of intervention can do better. In concert with other colleagues whose areas of expertise span the relevant fields, I have described (de Grey et al. 2002, 2004) a strategy built around the actual *repair* (not just retardation of accumulation) of age-related molecular and cellular damage—consisting of just seven major categories of ‘rejuvenation therapy’ (Table 1)—that appears technically feasible and, by its nature, is indefinitely extensible to greater life spans without recourse to further conceptual breakthroughs.

**Table 1:**
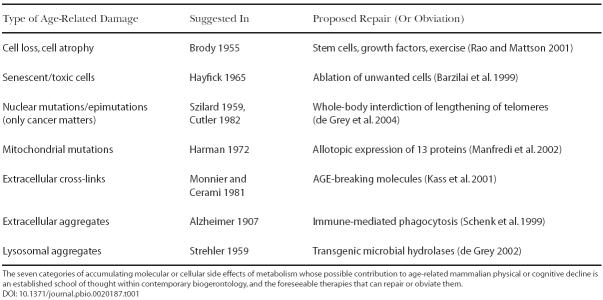
Strategies for Engineered Negligible Senescence

The seven categories of accumulating molecular or cellular side effects of metabolism whose possible contribution to age-related mammalian physical or cognitive decline is an established school of thought within contemporary biogerontology, and the foreseeable therapies that can repair or obviate them

The second oversight made both by the contributors to *Coping with Methuselah* and by other commentators is demographic. Life expectancy is typically defined in terms of what demographers call a period survival curve, which is a purely artificial construction derived from the proportions of those of each age at the start of a given year who die during that year. The ‘life expectancy’ of the ‘population’ thus described is that of a hypothetical population whose members live all their lives with the mortality risk at each age that the real people of that age experienced in the year of interest. The remaining life expectancy of someone aged *N* in that year is more than this life expectancy minus *N* for two reasons: one mathematical (what one actually wants, roughly, is the age to which the probability of survival is half that of survival to *N*) and one biomedical (mortality rates at each age, especially advanced ages, tend to fall with time). My spirits briefly rose on reading Aaron and Harris's explicit statement (p. 69) of the latter reason. Unfortunately, they didn't discuss what would happen if age-specific mortality rates fell by more than 2% per year. An interesting scenario was thus unexplored: that in which mortality rates fall so fast that people's *remaining* (not merely total) life expectancy increases with time. Is this unimaginably fast? Not at all: it is simply the ratio of the mortality rates at consecutive ages (in the same year) in the age range where most people die, which is only about 10% per year. I term this rate of reduction of age-specific mortality risk ‘actuarial escape velocity’ (AEV), because an individual's remaining life expectancy is affected by aging and by improvements in life-extending therapy in a way qualitatively very similar to how the remaining life expectancy of someone jumping off a cliff is affected by, respectively, gravity and upward jet propulsion (Figure 1).

**Figure 1:**
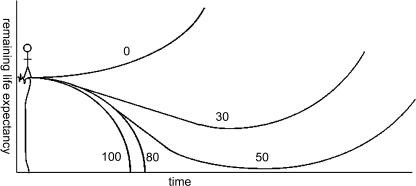
Physical and Actuarial Escape Velocities Remaining life expectancy follows a similar trajectory whether one walks off a cliff or merely ages: the time scales differ, but one's prognosis worsens with time. Mild mitigation of this (whether by jet propulsion or by rejuvenation therapies) merely postpones the outcome, but sufficiently aggressive intervention overcomes the force of gravity or frailty and increasingly distances the individual from a sticky end. Numbers denote plausible ages, at the time first-generation rejuvenation therapies arrive, of people following the respective trajectories.

The escape velocity cusp is closer than you might guess. Since we are already so long lived, even a 30% increase in healthy life span will give the first beneficiaries of rejuvenation therapies another 20 years—an eternity in science—to benefit from second-generation therapies that would give another 30%, and so on ad infinitum. Thus, if first-generation rejuvenation therapies were universally available and this progress in developing rejuvenation therapy could be indefinitely maintained, these advances would put us beyond AEV. Universal availability might be thought economically and sociopolitically implausible (though that conclusion may be premature, as I will summarise below), so it's worth considering the same question in terms of life-span *potential* (the life span of the luckiest people). Figure 1 again illustrates this: those who get first-generation therapies only just in time will in fact be unlikely to live more than 20–30 years more than their parents, because they will spend many frail years with a short remaining life expectancy (i.e., a high risk of imminent death), whereas those only a little younger will never get that frail and will spend rather few years even in biological middle age. Quantitatively, what this means is that if a 10% per year decline of mortality rates at all ages is achieved and sustained indefinitely, then the first 1000-year-old is probably only 5–10 years younger than the first 150-year-old.

The third oversight that I observe in contemporary commentaries on life extension, among which *Coping with Methuselah* is representative, is the most significant because of its urgency. First-generation rejuvenation therapies, whenever they arrive, will surely build on a string of prior laboratory achievements. Those achievements, it seems to me, will have progressively worn down humanity's evidently desperate determination to close its eyes to the prospect of defeating its foremost remaining scourge anytime soon. The problem (if we can call it that) is that this wearing-down may have been completed long before the rejuvenation therapies arrive. There will come an advance—probably a single laboratory result—that breaks the camel's back and forces society to abandon that denial: to accept that the risk of getting one's hopes up and seeing them dashed is now outweighed by the risk of missing the AEV boat by inaction. What will that result be? I think a conservative guess is a trebling of the remaining life span of mice of a long-lived strain that have reached two-thirds of their normal life span before treatment begins. This would possess what I claim are the key necessary features: a big life extension, in something furry and not congenitally sick, from treatment begun in middle age.

It is the prospect of AEV, of course, that makes this juncture so pivotal. It seems quite certain to me that the announcement of such mice will cause huge, essentially immediate, society-wide changes in lifestyle and expenditure choices—in a word, pandemonium—resulting from the anticipation that extreme human life extension might arrive soon enough to benefit people already alive. We will probably not have effective rejuvenation therapies for humans for at least 25 years, and it could certainly be 100 years. But given the present status of the therapies listed in Table 1, we have, in my view, a high probability of reaching the mouse life extension milestone just described (which I call ‘robust mouse rejuvenation’) within just *ten* years, given adequate and focused funding (perhaps $100 million per year). And nobody in *Coping with Methuselah* said so. This timeframe could be way off, of course, but as Wade notes (p. 57), big advances often occur much sooner than most experts expect. Even the most obvious of these lifestyle changes—greater expenditure on traditional medical care, avoidance of socially vital but risky professions—could severely destabilise the global economy; those better versed in economics and sociology than I would doubtless be even more pessimistic about our ability to negotiate this period smoothly. Overpopulation, probably the most frequently cited drawback of curing aging, could not result for many decades, but the same cannot be said for breadth of access irrespective of ability to pay: in a post-9/11 world, restricted availability of rejuvenation therapies resembling that seen today with AIDS drugs would invite violence on a scale that, shall we say, might be worth trying to avoid.

Am I, then, resigned to a future in which countless millions are denied many decades of life by our studied reluctance to plan ahead today? Not quite. The way out is pointed to in Lee and Tuljapurkar's (1997) graph of the average wealth consumed and generated by an individual as a function of age, reproduced in *Coping with Methuselah* (p. 143). Once AEV is achieved, there will be no going back: rejuvenation research will be intense forever thereafter and will anticipate and remedy the life-threatening degenerative changes appearing at newly achieved ages with ever-increasing efficacy and lead time. This will bring about the greatest economic change of all in society: the elimination of retirement benefits. Retirement benefits are for frail people, and there won't *be* any frail people. The graph just mentioned amply illustrates how much wealth will be released by this. My hope, therefore, is that once policy makers begin to realise what's coming they will factor in this eventual windfall and allocate sufficient short-term resources to make the period of limited availability of rejuvenation therapies brief enough to prevent mayhem. This will, however, be possible only if such resources begin to be set aside long enough in advance—and we don't know how long we have.

## Abbreviations

AEV: actuarial escape velocity
